# Transcriptomic Evidence of Immune–Tumor Uncoupling Defines a High-Risk State in Uterine Corpus Endometrial Carcinoma

**DOI:** 10.3390/ijms27104170

**Published:** 2026-05-07

**Authors:** Chia-Hung Chen, Hui-Ju Kao, Chen-Lin Yu, Tzu-Hsiang Weng, Tsung-Tao Huang, Kai-Yao Huang, Shun-Long Weng

**Affiliations:** 1 Department of Medical Research, Hsinchu MacKay Memorial Hospital, Hsinchu 30071, Taiwan; steven.l862@mmh.org.tw (C.-H.C.); hjk.l908@mmh.org.tw (H.-J.K.); l204.l204@mmh.org.tw (C.-L.Y.); 2 Department of Medical Research, Hsinchu Municipal MacKay Children’s Hospital, Hsinchu 30068, Taiwan; 3 Department of Obstetrics and Gynecology, MacKay Memorial Hospital, Taipei 10449, Taiwan; weng.3256@mmh.org.tw; 4 National Center for Instrumentation Research, National Institutes of Applied Research, Hsinchu 30076, Taiwan; tthuang@niar.org.tw; 5 Department of Medicine, MacKay Medical University, New Taipei 25245, Taiwan; 6 Institute of Biomedical Sciences, MacKay Medical University, New Taipei 25245, Taiwan; 7 Department of Biological Science and Technology, College of Engineering Bioscience, National Yang Ming Chiao Tung University, Hsinchu 30068, Taiwan; 8 Department of Obstetrics and Gynecology, Hsinchu MacKay Memorial Hospital, Hsinchu 30071, Taiwan; 9 Department of Obstetrics and Gynecology, Hsinchu Municipal MacKay Children’s Hospital, Hsinchu 30068, Taiwan

**Keywords:** uterine corpus endometrial carcinoma, immune–tumor uncoupling, transcriptomic signature, tumor microenvironment, angiogenesis, cell cycle

## Abstract

This study aimed to develop and validate a transcriptomic risk signature for uterine corpus endometrial carcinoma (UCEC) and to investigate whether the identified prognostic program reflects immune–tumor uncoupling within the tumor microenvironment. Using transcriptomic data from The Cancer Genome Atlas (TCGA) UCEC cohort, we identified a 28-gene transcriptomic signature defining a high-risk state. The derived risk score robustly stratified patients into distinct survival groups and remained an independent predictor of overall survival after adjustment for clinical covariates. Functional analyses revealed that high-risk tumors are characterized by a distinct immune–tumor uncoupling phenotype, in which interferon-gamma (IFNG)-associated inflammatory signaling is preserved but fails to translate into effective antitumor immune activity. Specifically, effector immune programs, including CD8 T cell-related signatures and cytotoxic activity, were consistently reduced despite elevated IFNG-associated signaling, indicating a functional discordance between immune activation and immune execution rather than classical T cell exhaustion. In parallel, high-risk tumors exhibited consistently elevated cell cycle and DNA repair-associated transcriptional programs, suggesting that proliferative and stress-adaptive mechanisms represent dominant drivers of poor prognosis. External assessment in an independent GEO cohort (GSE17025) demonstrated consistent associations between signature activity, tumor status, and histological grade, supporting the reproducibility of the underlying transcriptional program at the biological and clinicopathological level. Collectively, this study provides transcriptomic evidence for a previously underappreciated immune–tumor uncoupling state in UCEC and highlights the importance of integrating immune signaling and tumor-intrinsic programs to understand disease progression.

## 1. Introduction

Uterine corpus endometrial carcinoma (UCEC) represents the major histological subtype of corpus uteri cancer and is among the most common gynecologic malignancies worldwide. UCEC exhibits substantial biological heterogeneity, resulting in marked variability in clinical outcomes [1,2]. In the IARC Global Cancer Observatory, endometrial cancer is largely represented within the category of corpus uteri cancer, which accounted for approximately 420,368 new cases and 97,723 deaths globally in 2022, with an estimated 5-year prevalence of approximately 1.59 million cases. Moreover, GLOBOCAN Cancer Tomorrow projections estimate that the annual number of new corpus uteri cancer cases will increase to approximately 676,296 by 2050, corresponding to an approximately 60% increase from 2022 [3,4]. These epidemiological data underscore the growing global burden of endometrial cancer and support the need for improved molecular prognostic tools. Although many patients are diagnosed at an early stage and achieve favorable prognosis following standard surgical management, a clinically meaningful subset experience disease recurrence or progression despite apparently low-risk clinicopathological features [5,6]. This discordance between conventional clinicopathological risk stratification and clinical outcome highlights the limitations of staging- and histology-based frameworks and underscores the need for molecular stratification strategies that more accurately reflect underlying tumor biology and prognostic risk [7,8].

Advances in transcriptomic profiling have enabled the development of numerous gene expression-based prognostic signatures for UCEC and other solid tumors [9,10]. These molecular models have demonstrated prognostic value complementary to established clinical parameters [11]. However, many reported signatures primarily emphasize the optimization of predictive performance, while the biological programs represented by these models—and their relationships to immune-related and tumor-intrinsic transcriptional processes—often remain insufficiently characterized [12,13].

From a methodological perspective, several challenges persist in current prognostic modeling strategies for UCEC. Many studies rely on survival-driven feature selection pipelines or focus on single-dimensional biological axes, such as immune-related gene sets, without systematically integrating transcriptional programs associated with tumor proliferation, vascular adaptation, or genomic maintenance. Moreover, limited attention has been paid to whether selected prognostic genes reflect coordinated biological states or represent collections of statistically associated markers lacking functional coherence [13,14].

The tumor immune microenvironment has emerged as an important determinant of cancer progression and therapeutic response [15]. In UCEC, immune infiltration and immune-associated gene expression patterns have been linked to clinical outcomes, fostering the assumption that impaired antitumor immunity or immune evasion underlies poor prognosis [16,17]. Nevertheless, accumulating evidence across multiple cancer types indicates that immune-related transcriptional signals do not uniformly translate into effective antitumor immune responses or favorable survival outcomes. Instead, immune signaling may reflect context-dependent or functionally constrained immune states, highlighting the complexity of immune–tumor interactions and challenging simplified interpretations of immune activation signatures [15,18].

In parallel, tumor-intrinsic transcriptional programs—including cell cycle dysregulation, DNA damage response, angiogenesis, and epithelial–mesenchymal transition—have been implicated in aggressive tumor behavior and resistance to therapy [7,19,20]. These processes may act independently of, or in coordination with, immune-associated mechanisms to shape disease trajectory. However, the relative contributions and integration of immune-related and tumor-intrinsic programs within prognostic gene signatures for UCEC remain incompletely defined.

Beyond predictive accuracy, an additional analytical challenge lies in balancing prognostic performance with biological interpretability. While penalized regression and multivariable modeling approaches are effective for identifying prognostic gene sets, the resulting signatures are frequently treated as black-box models [13], as emphasized in recent methodological reviews [21]. Systematic functional annotation and integrative downstream analyses are therefore essential to bridge statistical prediction with biological understanding.

Several transcriptomic prognostic signatures have been proposed for UCEC, and many have demonstrated potential value for risk stratification. However, most existing models primarily emphasize predictive performance, whereas the biological interpretation of the inferred risk state remains incompletely characterized. In particular, how adverse transcriptomic risk states reflect the balance between immune activation, immune effector function, and tumor-intrinsic proliferative programs remain insufficiently understood. Therefore, beyond constructing another prognostic gene signature, there is a need to identify biologically interpretable transcriptomic states that may explain why certain UCEC tumors exhibit poor clinical outcomes despite evidence of inflammatory immune signaling.

In this study, we aimed to develop and validate a transcriptomic risk signature for UCEC and to determine whether the resulting high-risk state reflects a biologically interpretable pattern of immune–tumor uncoupling. Here, immune–tumor uncoupling is defined as a transcriptomic state in which IFNG-associated inflammatory signaling is preserved or elevated, but downstream effector immune programs, including CD8 T cell-related and cytotoxic activity signatures, are not correspondingly activated. By integrating survival modeling, functional enrichment, immune signature scoring, and external clinicopathological assessment, this study sought to move beyond risk prediction alone and to characterize a biologically meaningful transcriptomic state associated with poor prognosis in UCEC.

## 2. Results

### 2.1. Study Design and Analytical Workflow

**Figure 1:**
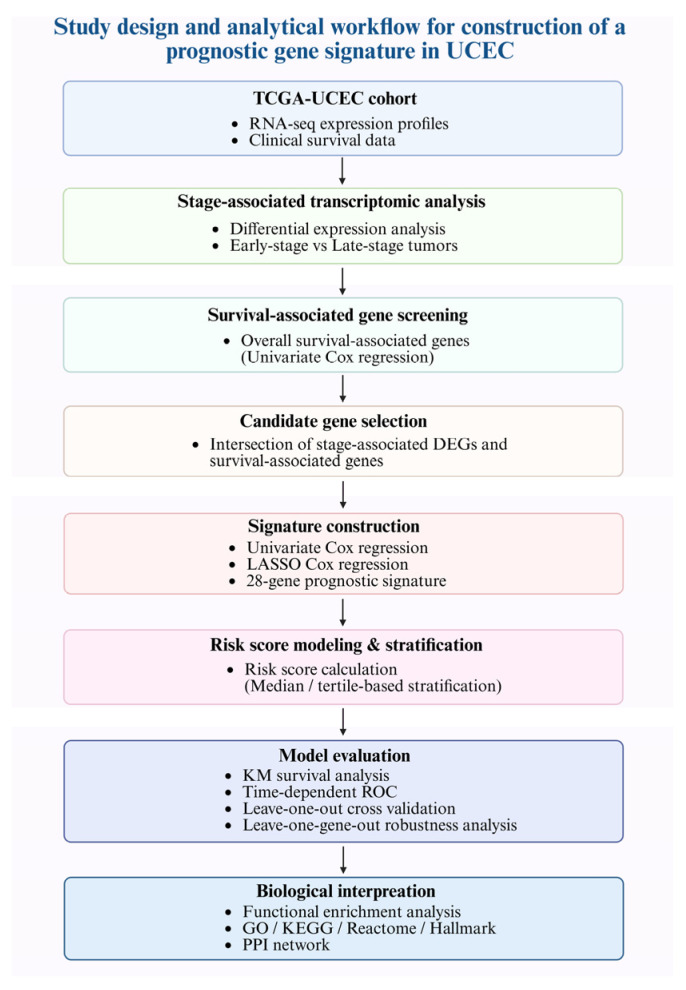
Study design and analytical workflow for identification of a high-risk transcriptomic signature in UCEC. Schematic overview of the analytical strategy used in the TCGA-UCEC cohort, including stage-based differential expression analysis, survival-associated gene screening, intersection-based candidate selection, LASSO Cox modeling, risk score stratification, performance evaluation, and downstream biological interpretation. Created in BioRender. Liao, K.W. (2026). https://BioRender.com/9ow4l8z (accessed on 4 May 2026) (Agreement number: IW29OP2H3A).

To provide an overview of the analytical strategy, a schematic workflow of the study design is shown in Figure 1. This study established an integrative transcriptomic framework to identify a high-risk transcriptomic signature/state in UCEC based on RNA-seq data and clinical survival information from the TCGA-UCEC cohort. Briefly, stage-associated differential expression analysis and survival-associated gene screening were performed in parallel to identify candidate genes related to disease progression and patient prognosis. The intersection of these gene sets was used to define a pool of biologically and clinically relevant candidates. These candidates were subsequently subjected to regression-based modeling to construct a multigene prognostic signature. The resulting risk model was further evaluated for prognostic performance, followed by downstream functional enrichment and network analyses to characterize the underlying biological programs associated with risk stratification. Detailed analytical procedures are described in the Section 4.

### 2.2. Identification of Stage- and Survival-Linked Genes Defining a 28-Gene High-Risk Transcriptional Signature

To characterize transcriptomic alterations associated with disease progression in UCEC, stage-based differential expression analysis was performed by comparing early-stage and late-stage tumors. This analysis identified a set of stage-associated differentially expressed genes, reflecting transcriptional changes accompanying tumor advancement (Figure S1). These genes were retained as biologically informed candidates for subsequent prognostic modeling. In parallel, genes associated with overall survival were identified using univariate Cox regression analysis. The intersection of stage-associated and survival-associated gene sets yielded 281 core candidate genes that were simultaneously linked to tumor progression and patient outcome and were carried forward for subsequent prognostic modeling. This integrative filtering step enriched for genes with both biological relevance and prognostic significance, reducing the likelihood of spurious associations driven solely by survival correlations.

Subsequent multistep filtering and penalized modeling identified a 28-gene transcriptional signature associated with a high-risk UCEC state. Cross-validation analysis demonstrated stable coefficient behavior across penalization paths (Figure S2), supporting the robustness of feature selection. Exploratory analyses, including hierarchical clustering and heatmap visualization, revealed distinct expression patterns of the selected genes across patients, consistent with the effective stratification of tumors according to prognostic risk. Together, these results establish a robust 28-gene signature that forms the basis for subsequent performance evaluation and biological interpretation.

### 2.3. Risk Stratification Defined by the 28-Gene Transcriptional Signature

**Figure 2:**
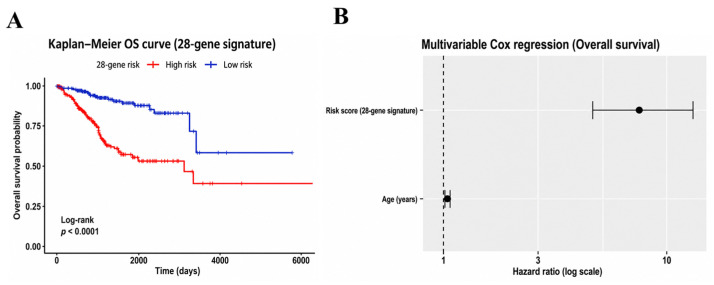
Prognostic stratification defined by the 28-gene transcriptional signature in the TCGA-UCEC cohort. (**A**) Kaplan–Meier overall survival (OS) curves for patients stratified into low- and high-risk groups based on the median risk score. Survival differences were evaluated using the log-rank test. (**B**) Multivariable Cox proportional hazards analysis for overall survival adjusting for age. Forest plot showing hazard ratios (HRs) and 95% confidence intervals for the risk score and age. The dashed vertical line indicates a hazard ratio (HR) of 1.0, and dots represent estimated HR values with corresponding 95% confidence intervals.

Based on the 28-gene high-risk transcriptional signature, a risk score was calculated for each patient as a weighted sum of gene expression levels derived from Cox regression coefficients. Patients were dichotomized into high-risk and low-risk groups using the median risk score as the cutoff. Kaplan–Meier survival analysis revealed a clear separation between the two groups, with high-risk patients exhibiting significantly poorer overall survival than low-risk patients (log-rank test, *p* < 0.0001; Figure 2A). Consistently, hierarchical clustering based on the expression profiles of the 28-gene signature showed distinct expression patterns between risk groups (Figure S3).

To assess whether the risk score reflected a continuous survival gradient, patients were further stratified into low-, intermediate-, and high-risk tertiles. A stepwise decrease in overall survival probability was observed across increasing risk categories, with the high-risk tertile showing the poorest prognosis and the intermediate-risk group displaying outcomes between the two extremes (Figure S4). These results support the robustness of the risk score as a continuous prognostic indicator.

### 2.4. Cox Regression Analyses of the Risk Score and Individual Signature Genes

Univariate Cox regression analysis was performed to evaluate the prognostic relevance of the 28-gene signature-derived risk score. The risk score was strongly associated with overall survival (HR = 8.14, 95% CI 5.34–12.40, *p* = 1.67 × 10^−22^), indicating a substantially increased risk of mortality among patients with higher scores. To assess the contribution of individual components within the signature, univariate Cox regression analysis was also conducted for each of the 28 genes. The corresponding hazard ratios, confidence intervals, and significance levels are summarized in Table 1, and their overall distribution is shown in Figure S5. Most genes exhibited hazard ratios deviating from unity, supporting their individual associations with overall survival. Together, these results indicate that the prognostic performance of the 28-gene signature reflects coordinated contributions from multiple genes rather than dependence on a single dominant predictor.

### 2.5. The Risk Score Is an Independent Prognostic Factor

To determine whether the prognostic value of the 28-gene signature-derived risk score was independent of established clinical variables, multivariable Cox proportional hazards regression analysis was performed. After adjustment for age, the risk score remained significantly associated with overall survival, indicating that the signature provides prognostic information beyond conventional clinical factors (Figure 2B; Table S1). In the multivariable model, higher risk scores were associated with a markedly increased hazard of death, whereas age showed a comparatively modest effect. Additional multivariable analyses incorporating FIGO stage and tumor grade, presented in the Supplementary Materials (Figure S6), further confirmed that the 28-gene risk score retained statistical significance after adjustment, supporting its prognostic value beyond standard clinicopathological stratification.

### 2.6. Predictive Performance, Calibration, and Robustness of the 28-Gene Signature

**Figure 3:**
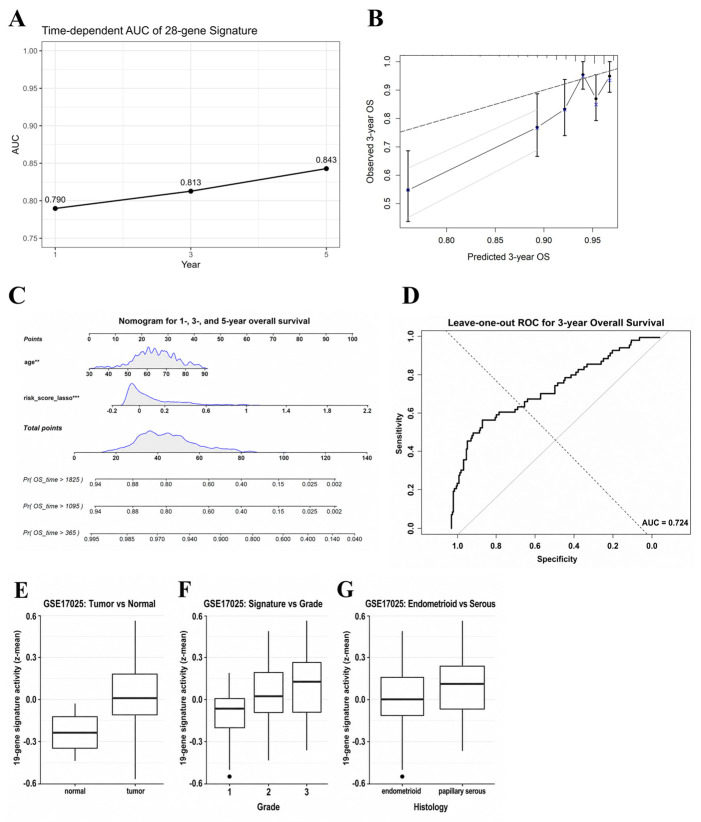
Predictive performance, internal robustness, and external biological/clinicopathological assessment of the 28-gene prognostic signature. (**A**) Time-dependent ROC analysis for 1-, 3-, and 5-year overall survival in the TCGA-UCEC cohort. (**B**) Calibration plot for three-year overall survival based on the multivariable model integrating the risk score and age. In the calibration plot, the black solid line represents the model calibration curve, the gray diagonal line indicates the ideal reference line, and error bars represent 95% confidence intervals. (**C**) Nomogram for the prediction of 1-, 3-, and 5-year overall survival. In the nomogram, ** and *** indicate *p* < 0.01 and *p* < 0.001, respectively. (**D**) Leave-one-out cross-validation (LOO-CV) ROC curve for 3-year overall survival. In the ROC plot, the black curve represents the leave-one-out cross-validation ROC curve, and the gray diagonal line indicates random classification performance. (**E**) Comparison of signature activity between tumor and normal tissues in the independent GSE17025 cohort. (**F**) Distribution of signature activity across tumor grades. (**G**) Comparison of signature activity between histological subtypes. Signature activity in the external cohort was calculated using available signature genes on the microarray platform. Statistical significance was assessed using Wilcoxon rank-sum or Kruskal–Wallis tests.

The predictive performance of the 28-gene signature was evaluated using time-dependent ROC analysis. As shown in Figure 3A, the risk score demonstrated stable discriminatory ability for overall survival, with AUC values of approximately 0.79, 0.81, and 0.84 at 1, 3, and 5 years, respectively, indicating consistent prognostic accuracy across clinically relevant time points. Model calibration was examined by comparing predicted and observed survival probabilities. The 3-year calibration plot showed close agreement between predicted and actual outcomes, with data points aligning near the ideal reference line (Figure 3B). A nomogram integrating age and the 28-gene risk score was constructed to facilitate individualized prediction of 1-, 3-, and 5-year overall survival (Figure 3C), highlighting the dominant contribution of the risk score relative to age. Model robustness was assessed using sample-level leave-one-out cross-validation for three-year overall survival, in which each patient was iteratively excluded and the model refit on the remaining samples, yielding an AUC of 0.724 (Figure 3D). In a limited number of iterations, time-dependent AUCs could not be estimated due to insufficient event information; these cases were treated as missing values and did not materially affect the robustness assessment.

Consistently, leave-one-gene-out sensitivity analysis showed that the 3-year AUC remained stable after removal of any single gene from the signature (Table S2), indicating that prognostic performance was not driven by a dominant component. Together, these results support the stability, discrimination, and calibration of the 28-gene signature as a prognostic tool in UCEC.

### 2.7. External Biological and Clinicopathological Assessment of Signature Activity in an Independent Cohort

To evaluate whether the transcriptional program represented by the 28-gene signature was reproducible beyond the TCGA cohort, we performed an external biological and clinicopathological assessment using the independent GSE17025 dataset. Because GSE17025 was generated using a microarray platform, only a subset of the 28 signature genes was available, and signature activity was therefore estimated using the available genes rather than the complete 28-gene model. After probe-to-gene mapping, 19 of the 28 signature genes were retained, and signature activity was calculated without cross-cohort model refitting. As shown in Figure 3E, signature activity was significantly higher in tumor tissues than in normal endometrium (Wilcoxon rank-sum test, *p* < 0.001), supporting its association with malignant transformation. Signature activity also increased stepwise with tumor grade (Kruskal–Wallis test, *p* = 0.0037), indicating a consistent relationship with histological aggressiveness (Figure 3F). Although differences between endometrioid and papillary serous subtypes were not statistically significant (Wilcoxon rank-sum test, *p* = 0.24), the overall distribution pattern was concordant with observations in the TCGA cohort (Figure 3G). Together, these results indicate that the transcriptional program captured by the 28-gene signature shows reproducible associations with tumor-related clinicopathological features across independent cohorts, despite reduced gene coverage inherent to the microarray platform. These findings should therefore be interpreted as external biological and clinicopathological support for the reproducibility of the signature-associated transcriptional program, rather than as full external prognostic validation of the complete 28-gene model, because only 19 signature genes were available on the microarray platform and survival-based assessment of the complete model was not feasible.

### 2.8. Functional Enrichment Analysis of the 28-Gene Prognostic Signature

**Figure 4:**
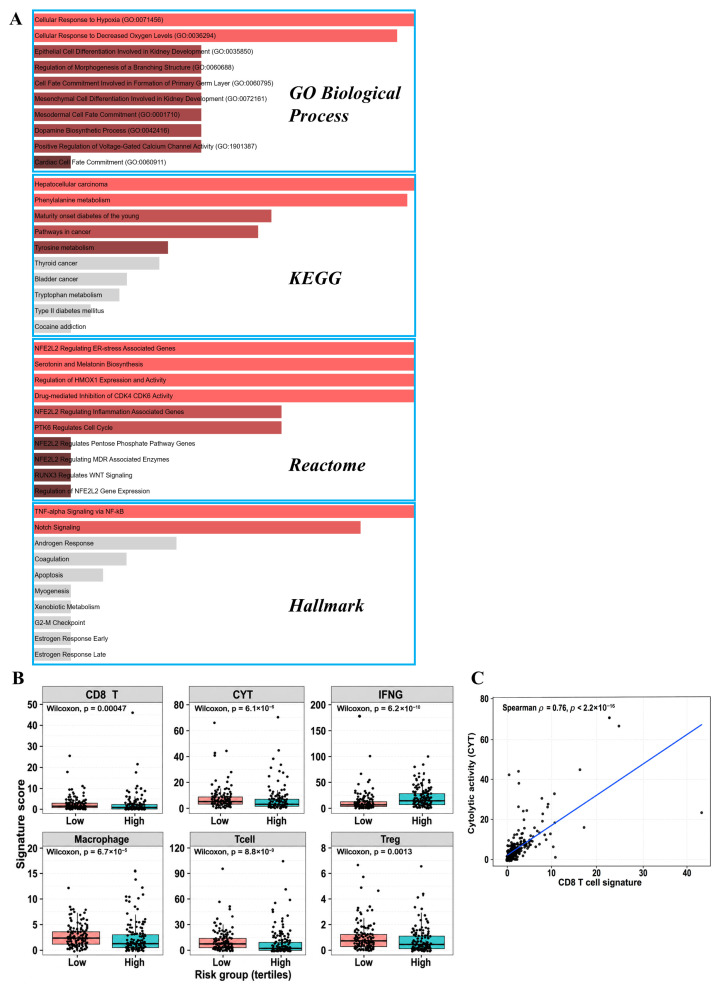
Functional enrichment and immune-related transcriptomic signatures associated with the 28-gene prognostic model. Enrichment analyses identify pathway-level programs associated with differential transcriptional states, whereas sample-level scoring evaluates the relative activity of selected immune or tumor-intrinsic programs across individual tumors. (**A**) Functional enrichment analysis showing representative enriched terms across Gene Ontology (GO) Biological Process, KEGG, Reactome, and Hallmark databases for genes associated with the 28-gene signature-defined high-risk state. (**B**) Comparison of immune-related transcriptomic signature scores between low- and high-risk groups defined by tertile stratification, including CD8 T cell signatures, cytotoxic activity (CYT), IFNG signaling, macrophage-related signatures, total T cell signatures, and regulatory T cell (Treg) signatures. (**C**) Correlation between CD8 T cell signature scores and cytotoxic activity (CYT), assessed using Spearman correlation analysis.

To elucidate the biological processes underlying the prognostic relevance of the 28-gene signature, the selected genes were first annotated according to their reported functional roles and pathway involvement (Table S3), followed by functional enrichment analyses across multiple knowledge bases, including Gene Ontology (GO), KEGG, Reactome, and Hallmark gene sets. Across these databases, the 28-gene signature was consistently enriched in biological processes related to inflammatory signaling, hypoxia response, epithelial differentiation and epithelial–mesenchymal transition (EMT), metabolic reprogramming, and cell cycle-associated programs (Figure 4A). GO analysis highlighted significant enrichment in hypoxia-related processes, including cellular response to hypoxia and decreased oxygen levels, suggesting a link between the signature and hypoxic tumor microenvironments. Additional GO terms related to cell fate commitment and epithelial differentiation further implicated developmental programs relevant to tumor progression. KEGG pathway analysis identified enrichment in cancer-associated pathways as well as amino acid metabolism, indicating that the signature captures both oncogenic signaling and metabolic adaptation. Reactome analysis further highlighted pathways associated with oxidative stress response and cell cycle regulation, including NFE2L2-mediated stress responses and CDK4/6-related proliferative control. Consistently, Hallmark enrichment analysis identified TNF-α signaling via NF-κB and Notch signaling, both of which are associated with inflammatory activation, tumor aggressiveness, and immune modulation. Collectively, these results suggest that the 28-gene signature captures a complex transcriptional landscape integrating inflammatory signaling, metabolic adaptation, and proliferation-related processes, providing a mechanistic basis for subsequent immune and tumor behavior analyses.

### 2.9. Immune Landscape Reveals Functional Immune–Tumor Uncoupling Associated with the 28-Gene Signature

Based on the enrichment patterns observed, immune-related transcriptional programs were further characterized to define the immune landscape associated with the 28-gene prognostic signature. Comparison of immune-related signature scores between the lowest- and highest-risk tertiles revealed a discordant immune phenotype. High-risk tumors exhibited significantly elevated IFNG-associated signaling, whereas multiple effector immune programs—including CD8 T cell-related signatures, cytotoxic activity (CYT), and overall T cell signatures—were significantly reduced compared with low-risk tumors (Wilcoxon rank-sum test, all *p* < 0.01; Figure 4B). Macrophage- and regulatory T cell-associated signatures were also reduced in the high-risk group, indicating attenuated immune-related transcriptional programs despite elevated inflammatory signaling. Correlation analysis demonstrated strong internal coherence among immune activation metrics, with a robust positive association between CD8 T cell signatures and cytotoxic activity (Spearman’s R = 0.76, *p* < 2.2 × 10^−16^; Figure 4C), supporting the coordinated nature of effector immune programs. Enrichment analyses and sample-level signature scoring should be interpreted as complementary rather than interchangeable approaches. Enrichment analyses identify pathway-level programs associated with differential transcriptional states, whereas sample-level signature scoring evaluates the relative activity of selected immune or tumor-intrinsic programs across individual tumors (Figure 4).

**Figure 5:**
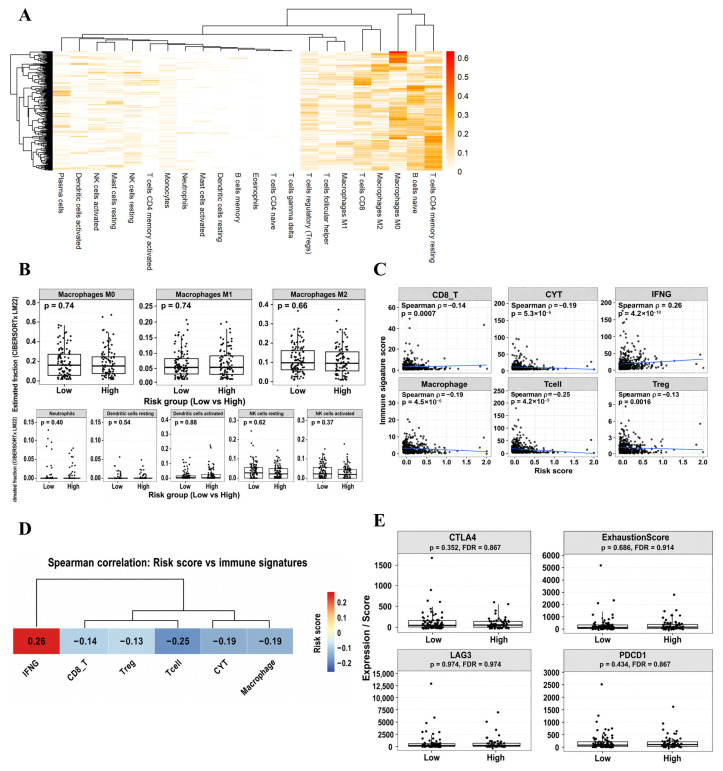
Immune landscape characterization associated with the 28-gene prognostic signature. (**A**) Heatmap showing LM22-estimated immune cell fractions across the TCGA-UCEC cohort using CIBERSORTx. (**B**) Comparison of innate immune cell subsets between low- and high-risk groups, including macrophage polarization states (M0, M1, and M2), neutrophils, dendritic cells, and NK cells. (**C**) Associations between continuous risk scores and immune-related transcriptomic signature scores, including CD8 T cell infiltration, cytotoxic activity (CYT), IFNG signaling, macrophage-related signatures, overall T cell signatures, and regulatory T cell (Treg) signatures. Spearman correlation coefficients are shown. (**D**) Heatmap of Spearman correlation coefficients between the risk score and immune-related transcriptomic signatures. (**E**) Comparison of selected immune checkpoint markers (*PDCD1*, *LAG3*, and *CTLA4*) between low- and high-risk groups.

To further investigate whether these transcriptional differences were associated with changes in immune cell composition, LM22-based CIBERSORTx deconvolution was performed. Immune cell fractions exhibited substantial inter-sample heterogeneity without a dominant clustering pattern (Figure 5A). Importantly, no significant differences were observed between risk groups in macrophage polarization states (M0, M1, and M2), neutrophils, dendritic cells, or NK cell subsets (Figure 5B), indicating that the observed immune transcriptional differences were not driven by changes in estimated immune cell abundance. Continuous risk score-based analyses further revealed inverse associations between risk scores and multiple effector immune signatures, whereas IFNG signaling showed a positive association with risk (Figure 5C,D). Assessment of classical T cell exhaustion markers (*PDCD1*, *LAG3*, and *CTLA4*) demonstrated no significant differences between risk groups, and composite exhaustion scores were likewise unchanged (Figure 5E). Taken together, these findings indicate that high-risk tumors are characterized by elevated IFNG-associated inflammatory signaling in the absence of coordinated effector immune activation, immune cell expansion, or classical exhaustion phenotypes. This pattern supports a functional dissociation between immune signaling and effective antitumor immune execution, consistent with an immune–tumor uncoupling state.

### 2.10. Tumor Behavior-Associated Transcriptional Programs Linked to Proliferative and Adaptive Dominance

**Figure 6:**
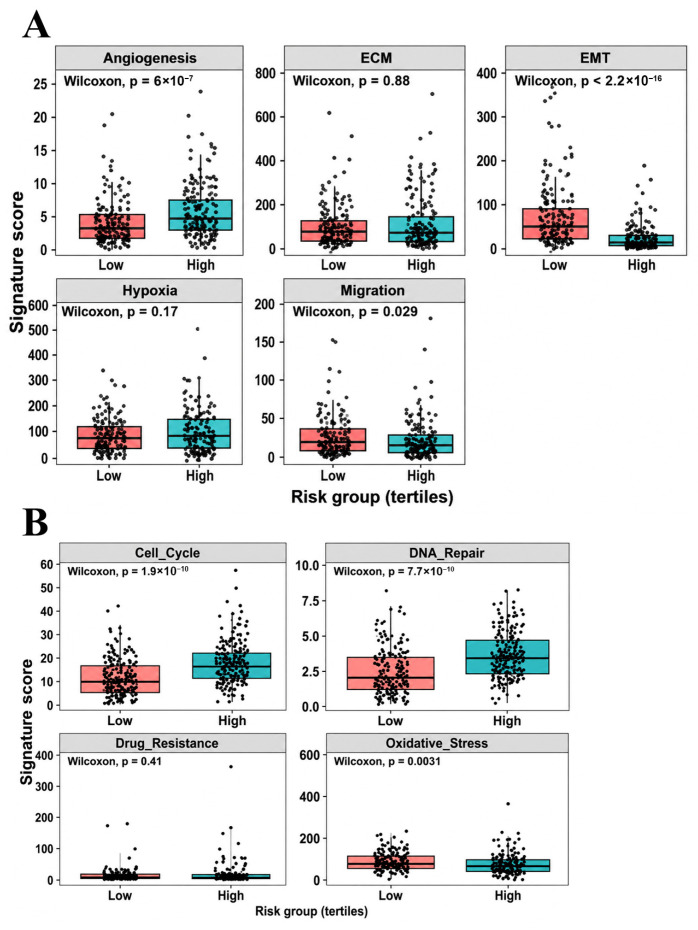
Tumor behavior-associated transcriptomic programs linked to prognostic risk. (**A**) Comparison of tumor behavior-associated transcriptomic signatures between low- and high-risk groups, including epithelial–mesenchymal transition (EMT), angiogenesis, extracellular matrix (ECM), hypoxia, and migration-related programs. (**B**) Comparison of proliferation- and stress-related transcriptomic signatures between risk groups, including cell cycle regulation, DNA repair, drug resistance, and oxidative stress. Group differences were evaluated using the Wilcoxon rank-sum test.

Given the enrichment of tumor progression-related pathways, tumor-intrinsic transcriptional programs were further analyzed to characterize the biological features associated with prognostic risk. EMT activity was quantified using a marker-based scoring approach integrating mesenchymal and extracellular matrix (ECM)-associated genes together with epithelial markers. EMT-related signature scores differed significantly between high- and low-risk tumors (Wilcoxon test, *p* < 2.2 × 10^−16^; Figure 6A), indicating risk-associated variation in EMT-related transcriptional features. However, these differences did not reflect a uniformly elevated EMT-driven program in high-risk tumors. Angiogenesis-related signatures were significantly enriched in the high-risk group (*p* = 6 × 10^−7^), while migration-related signatures showed more modest but significant differences (*p* = 0.029). In contrast, ECM- and hypoxia-associated signatures did not differ significantly between groups, suggesting that hypoxia-related enrichment at the gene level does not translate into a coherent sample-level transcriptional program.

Further analyses of proliferative and stress-related pathways revealed that high-risk tumors exhibited markedly elevated cell cycle activity and DNA repair-related signatures (both *p* < 1 × 10^−9^; Figure 6B), consistent with enhanced proliferative capacity and reinforced genomic maintenance mechanisms. Drug resistance-associated signatures did not differ significantly between risk groups, while oxidative stress-related signatures were modestly higher in the low-risk group. Collectively, these results indicate that high-risk tumors are characterized by enhanced angiogenesis and strong proliferative and DNA repair programs, rather than a classical EMT-dominated invasive phenotype. Together with the immune findings, these results support a model in which tumor-intrinsic proliferative and adaptive programs dominate over effective immune responses in high-risk UCEC.

### 2.11. Protein–Protein Interaction Network Analysis Reveals Coordinated Functional Modules Within the 28-Gene Signature

**Figure 7:**
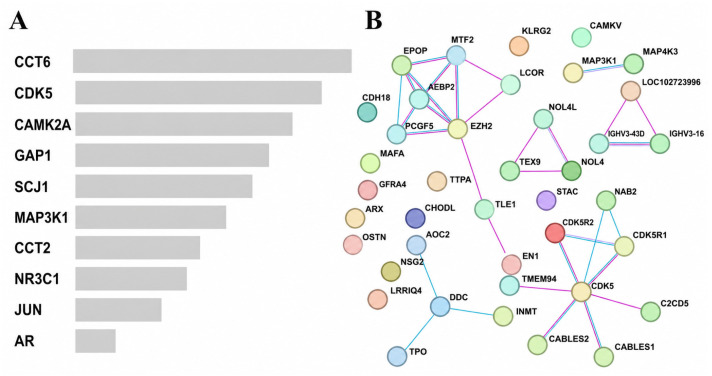
Network-level functional organization of the 28-gene prognostic signature. (**A**) Bar plot showing the top-ranked interacting partner genes identified by PPI network analysis based on the 28-gene signature, ranked by degree of connectivity. (**B**) Protein–protein interaction (PPI) network constructed using the 28-gene signature and their first-order interacting partners from the STRING database. Nodes represent genes or proteins, and edges indicate known or predicted interactions. Node colors indicate network modules identified by clustering. The STRING interaction confidence score cutoff was set at 0.300.

To investigate whether the 28 prognostic genes are embedded within a biologically connected protein network, PPI analysis was conducted using the STRING database. Analysis of first-order interacting partners revealed that several non-signature genes exhibited high degrees of connectivity within the reconstructed network, including *CCT6*, *CAMK2A*, *MAP3K1*, and additional stress- or signaling-related molecules (Figure 7A), highlighting a network context enriched for protein folding, signal transduction, and cellular stress–response processes. Visualization of the PPI network demonstrated that multiple signature genes occupy topologically central positions and form interconnected functional modules (Figure 7B). Core signature genes such as *EZH2* and *CDK5*, together with their regulatory partners *CDK5R1* and *CDK5R2*, were located at the center of a densely connected module related to cell cycle regulation and transcriptional control. Additional modules involved signaling regulators such as *MAP3K1*, indicating that certain genes function both as signature components and as connectors within broader signaling networks.

The PPI network exhibited a modular organization consistent with enrichment results, including epigenetic and transcriptional regulators (*EZH2*, *EPOP*, *AEBP2*, and *PCGF5*) and a *CDK5*-centered module involving *CDK5R1*, *CDK5R2*, *CABLES1*, and *CABLES2*, supporting coordinated roles in chromatin regulation, cell cycle control, and stress-adaptive signaling.

**Figure 8:**
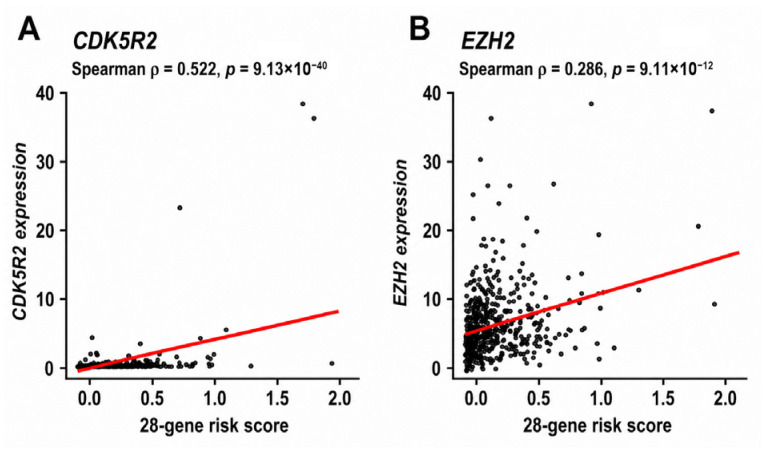
Association between expression of PPI-identified hub genes and the 28-gene risk score in the TCGA-UCEC cohort. Scatter plots showing the relationships between the 28-gene risk score and the expression levels of representative hub genes identified from PPI network analysis, including (**A**) *CDK5R2* and (**B**) *EZH2*. Each point represents an individual tumor sample. Spearman correlation coefficients (ρ) and corresponding *p* values are shown.

To provide transcript-level support for the PPI-identified hubs, we examined the associations between representative module genes and the 28-gene risk score and overall survival in the TCGA-UCEC cohort (Table 2 and Figure 8). *EZH2* expression was positively correlated with the 28-gene risk score (Spearman ρ = 0.286, *p* = 9.1 × 10^−12^) and was higher in the high-risk group (Wilcoxon *p* = 1.7 × 10^−10^); higher *EZH2* expression was also associated with poorer overall survival (HR = 1.061, 95% CI 1.023–1.100; *p* = 0.0015). Notably, *CDK5R2* showed a strong positive association with the risk score (ρ = 0.522, *p* < 2.2 × 10^−16^) and adverse survival outcomes (HR = 1.087, 95% CI 1.049–1.126; *p* = 3.7 × 10^−6^), while *CDK5R1* also exhibited prognostic relevance in survival analysis (HR = 1.142, 95% CI 1.029–1.267; *p* = 0.012). In contrast, *CDK5* and *MAP3K1* did not display significant individual prognostic effects, indicating that not all topologically central genes contribute equally at the transcript level. Consistent with these findings, PPI network analysis highlighted *EZH2* and a *CDK5*-centered module as highly connected nodes linked to the 28-gene prognostic signature (Figure 7B).

Notably, the functional composition of these PPI modules was highly concordant with the pathways enriched in earlier analyses, including Hallmark cell cycle checkpoints, inflammatory and immune signaling, oxidative stress responses, and epithelial–mesenchymal transition. This convergence of pathway enrichment, signature-based functional scoring, and protein interaction network analysis provides convergent computational evidence supporting the biological coherence of the 28-gene prognostic signature. It is worth noting that several hub genes identified by network topology were not necessarily the strongest predictors in univariate survival analysis, underscoring that network centrality reflects functional coordination within oncogenic programs rather than individual prognostic dominance. Together, these findings suggest that the prognostic value of the 28-gene signature arises from integrated biological processes rather than isolated gene effects.

## 3. Discussion

**Figure 9:**
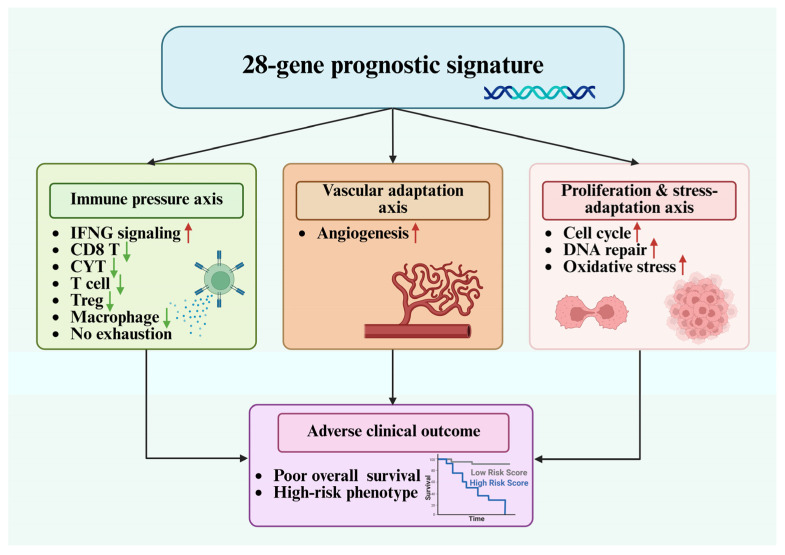
Conceptual model summarizing the major transcriptomic axes associated with the 28-gene prognostic signature in UCEC. Schematic overview integrating findings from enrichment, transcriptomic signature, and network analyses. Three major axes are depicted: IFNG-associated inflammatory signaling, vascular adaptation, and proliferation/stress-related programs. Created in BioRender. Liao, K.W. (2026). https://BioRender.com/cmtrac8 (accessed on 4 May 2026) (Agreement number: TR29OP2AFL).

To integrate the molecular features underlying the 28-gene prognostic signature, we constructed an integrative framework summarizing the major transcriptomic programs associated with adverse outcomes in UCEC (Figure 9). Rather than reflecting a single dominant process, the signature defines a coordinated high-risk transcriptomic state characterized by the coexistence of immune-associated inflammatory signaling and tumor-intrinsic adaptation programs. One possible interpretation is that preserved IFNG-associated signaling reflects inflammatory stimulation or immune recognition, whereas reduced CD8 T cell-related and cytotoxic programs suggest a failure of downstream effector immune execution. In this context, high-risk tumors may maintain an inflammatory transcriptional background while simultaneously acquiring tumor-intrinsic proliferative, DNA repair, and stress-adaptive programs that reduce the effectiveness of antitumor immunity. This dissociation may therefore represent a state in which immune activation signals are present but insufficient to produce coordinated cytotoxic immune activity. This interpretation is supported by risk-stratified functional analyses (Figure 4), which demonstrated preserved IFNG-related signaling alongside attenuated effector immune programs and enhanced proliferation- and stress-related pathways.

More broadly, transcriptomic profiles should be interpreted as dynamic biological snapshots that may be shaped by tissue-level stress responses, hypoxia-associated signaling, angiogenic remodeling, metabolic adaptation, and recovery-like processes. These dynamic processes may influence the apparent relationship between inflammatory signaling and effector immune activity, particularly when functional states are inferred from bulk transcriptomic data. Therefore, immune–tumor uncoupling should be regarded as a dynamic tumor-state phenotype rather than a fixed or fully resolved functional mechanism.

The main contribution of this study is not the introduction of a new statistical modeling algorithm, but the identification of a biologically interpretable transcriptomic risk state in UCEC. Although the analytical framework used established approaches, including differential expression analysis, Cox regression, and LASSO-based feature selection, the 28-gene signature was not interpreted solely as a predictive score. Instead, it was used to characterize a high-risk tumor state marked by immune–tumor uncoupling, attenuated effector immune activity, and enhanced tumor-intrinsic proliferative and DNA repair-associated programs. This biologically oriented interpretation distinguishes the present study from purely predictive transcriptomic signature studies. Importantly, this framework highlights a functional dissociation between immune signaling and effective antitumor immune execution, rather than classical immune-inflamed or immune-desert phenotypes [22,23,24]. Consistent tumor–normal separation and grade-associated trends observed in an independent cohort (GSE17025), despite reduced gene coverage, further support that this transcriptional program reflects conserved features of disease progression.

Previous transcriptome-based prognostic signatures in UCEC have primarily emphasized predictive performance metrics such as C-index or time-dependent AUC [14]. However, cross-study comparisons are limited by heterogeneity in cohorts, platforms, and analytical strategies. Many models rely on survival-driven feature selection, which may not capture biologically coherent or system-level programs, and often provide limited mechanistic insight [13,25,26]. In contrast, the present study identifies a biologically interpretable high-risk transcriptomic state, emphasizing robustness and integrative biological characterization rather than direct benchmarking.

Although the present study was based on tumor bulk transcriptomic data rather than germline genotyping, the identified transcriptional programs can be interpreted in the context of prior GWAS and genetic association studies of endometrial cancer. GWAS studies have identified multiple susceptibility loci for endometrial cancer, including regions near *CYP19A1*, *HNF1B*, *MYC*, *KLF5*, *AKT1*, *EIF2AK4*, and related candidate genes, implicating hormonal regulation, transcriptional control, cell proliferation, and oncogenic signaling in disease susceptibility. While these germline susceptibility loci are not expected to directly overlap with a tumor-derived prognostic expression signature, they provide complementary evidence that dysregulated proliferative and tumor-intrinsic programs are central to endometrial cancer biology. Consistent with this concept, our high-risk transcriptomic state was characterized by elevated cell cycle and DNA repair-associated programs, together with immune–tumor uncoupling. Therefore, the present findings should be viewed as complementary to GWAS-based susceptibility studies: GWAS highlights inherited risk architecture, whereas our transcriptomic analysis captures tumor-state programs associated with disease progression and prognosis.

At the immune level, high-risk tumors exhibited elevated IFNG-associated signaling but reduced effector immune programs, including CD8 T cell signatures, cytotoxic activity, and overall T cell-related transcriptional profiles. Notably, canonical exhaustion markers (*PDCD1*, *CTLA4*, and *LAG3*) and composite exhaustion scores did not differ between risk groups (Figure 5). These findings define an IFNG-high but effector-low immune context, indicating a functional disconnect between inflammatory signaling and immune execution. However, these findings should be interpreted as transcriptomic evidence of an immune–tumor uncoupling phenotype rather than direct functional proof of impaired antitumor immunity. This immune context is not consistent with classical T cell exhaustion [27,28], but instead reflects a state of chronic immune pressure and sustained interferon exposure, in which inflammatory signals persist without coordinated cytotoxic responses. Such IFN-γ-adapted states have been increasingly recognized as context-dependent immune phenotypes associated with functional constraint rather than effective tumor clearance [29]. In contrast, tumor-intrinsic programs emerged as dominant drivers of adverse outcomes. High-risk tumors showed coordinated upregulation of angiogenesis, cell cycle progression, and DNA repair pathways (Figure 4), indicating enhanced proliferative capacity and stress tolerance. These features likely outweigh any protective effects of inflammatory signaling, contributing to disease progression [30]. Notably, this aggressive phenotype was not associated with uniform activation of invasion-related programs. Although angiogenesis-related signatures were elevated, EMT- and migration-associated programs were not consistently activated, suggesting that poor prognosis is driven by selective engagement of proliferative and vascular pathways rather than global mesenchymal transition [31]. Similarly, although hypoxia-related terms were enriched at the annotation level, hypoxia-associated transcriptomic scores did not differ significantly between groups, indicating partial or context-dependent pathway involvement. This highlights the importance of distinguishing enrichment signals from coordinated functional activation when interpreting gene signatures [13,21].

At the systems level, PPI network analysis (Figure 7) identified regulatory hubs such as *EZH2*, an epigenetic regulator [32,33], and a *CDK5*-centered axis involving *CDK5R2* [34,35], supporting the biological coherence of the signature. Their association with higher risk scores further indicates that prognostic relevance arises from coordinated regulatory networks rather than individual genes. Collectively, these findings support a model in which the 28-gene signature defines a refined high-risk transcriptomic state, where tumor-intrinsic proliferative and vascular programs dominate over functionally constrained immune signaling. This selective, rather than global, pathway activation underscores a non-uniform biological basis of poor prognosis.

From an analytical perspective, this study highlights the value of integrative modeling strategies that capture coordinated gene behavior through multi-step approaches, including differential expression, survival screening, penalized modeling, and robustness assessment. From a clinical perspective, the risk score remained an independent prognostic factor after adjustment for stage and grade, suggesting additive value for refined risk stratification. Several limitations should be acknowledged. First, the present study was based on bulk transcriptomic mRNA-level data, and therefore cannot directly resolve protein-level regulation, spatial cellular organization, or functional immune activity within the tumor microenvironment. Second, external validation was performed using a microarray cohort with partial coverage of the 28-gene signature, and complete external survival validation of the full model was not feasible in this dataset. Nevertheless, the consistent associations between signature activity, tumor status, and histological grade support the biological and clinicopathological reproducibility of the underlying transcriptional program. Third, direct benchmarking against previously published UCEC prognostic signatures was not performed in the present study. Because existing models differ in gene composition, coefficient availability, transcriptomic platforms, preprocessing methods, and clinical endpoint definitions, rigorous head-to-head comparison would require harmonized datasets and standardized evaluation metrics. Therefore, while the present study emphasizes the biological interpretability of the 28-gene signature and its association with an immune–tumor uncoupling state, future studies will be required to determine its incremental predictive value relative to existing prognostic models. Fourth, a direct statistical correlation between the 28-gene risk signature and GWAS-derived polygenic risk scores could not be performed because matched germline genotype data and GWAS-derived risk scores were not available in the present analysis. Fifth, although the immune–tumor uncoupling phenotype was inferred from multiple transcriptomic analyses, functional experimental validation was not performed in the present study. Future studies integrating germline genetics, tumor transcriptomics, spatial profiling, proteomics, and experimental models may further clarify the biological mechanisms and clinical relevance of this transcriptomic risk state in UCEC.

In summary, poor prognosis in UCEC was associated with a transcriptomic tumor state characterized by increased proliferative and stress-adaptive programs alongside preserved inflammatory signaling but attenuated effector immune activity. This non-classical high-risk configuration supports the value of system-level transcriptomic analysis beyond performance-driven prognostic modeling and provides a framework for interpreting disease progression at the transcriptomic level. Further studies, including functional and experimental validation, are required to elucidate the underlying biological mechanisms and determine their clinical relevance.

## 4. Materials and Methods

### 4.1. Data Sources and Preprocessing

RNA sequencing (RNA-seq) expression profiles and corresponding clinical information for UCEC patients were obtained from The Cancer Genome Atlas (TCGA-UCEC) cohort, downloaded in December 2025. Only primary tumor samples with available overall survival (OS) data were included in downstream analyses. Patients lacking survival information or with incomplete clinical annotation were excluded.

Gene expression matrices were processed at the transcriptome-wide level. Gene identifiers were standardized to Ensembl gene IDs, and genes with consistently low expression across samples were filtered out prior to differential expression and survival analyses. All analyses were performed using R (version 4.5.2). Key analytical procedures were implemented using well-established R packages, including glmnet (v4.1-8) for LASSO Cox regression, survival (v3.5-7) for Cox proportional hazards modeling, and survivalROC (v1.0.3) for time-dependent receiver operating characteristic (ROC) analysis. Differential expression analyses were conducted using DESeq2 (v1.38.3), while data manipulation and visualization were performed using tidyverse (v2.0.0) and ggplot2 (v3.5.0). DESeq2 normalization was applied to raw count data to account for library size and compositional differences across samples [36]. No additional batch correction was performed, as all samples were derived from a single TCGA cohort and processed using a unified sequencing and bioinformatics pipeline.

Genes with consistently low expression across samples were excluded prior to downstream analyses to reduce noise and improve statistical robustness. Specifically, genes were retained if they showed detectable expression in a sufficient proportion of tumor samples, resulting in 20,318 expressed genes used for subsequent differential expression and survival analyses. For downstream survival modeling and risk score construction, count-based normalized expression values were used. FPKM and TPM values were not adopted, as these measures are less appropriate for cross-sample comparisons and regression-based survival analyses due to potential compositional bias. Instead, count-based normalization approaches were consistently applied throughout the study to ensure statistical robustness and comparability across samples. Gene-set-based signature scoring and downstream functional analyses were performed using custom R scripts in conjunction with publicly available gene signature resources.

### 4.2. Identification of Stage-Associated Differentially Expressed Genes

To identify genes associated with disease progression, patients were stratified according to pathological stage into early-stage and late-stage groups. Differential expression analysis was conducted by comparing normalized RNA-seq expression levels between these two groups. Genes exhibiting statistically significant expression differences were defined as stage-associated differentially expressed genes (DEGs).

The global distribution of DEGs was visualized using volcano plots, displaying log2 fold changes and false discovery rate (FDR)-adjusted *p*-values. Genes with an absolute log2 fold change ≥ 1 and a FDR < 0.05 were defined as differentially expressed.

### 4.3. Survival-Associated Gene Screening

To identify genes associated with patient prognosis, univariate Cox proportional hazards regression was performed for each gene using overall survival as the endpoint. Genes with statistically significant associations with OS were defined as survival-associated genes. Genes with a univariate Cox regression *p* value < 0.05 were considered survival-associated genes and retained for downstream analysis.

Candidate genes for prognostic model construction were obtained by intersecting stage-associated DEGs with survival-associated genes, thereby enriching for genes with both biological relevance to tumor progression and clinical relevance to patient outcome.

### 4.4. Construction of the Prognostic Gene Signature

Candidate genes were first evaluated using univariate Cox regression to estimate individual hazard ratios. To reduce multicollinearity and mitigate overfitting, least absolute shrinkage and selection operator (LASSO) Cox regression was applied using 10-fold cross-validation.

Two commonly used penalty parameters were evaluated: the minimum criteria (λ_min), which yields the lowest cross-validated partial likelihood deviance, and the one-standard-error criterion (λ_1se), which favors a more parsimonious model. In this study, λ_min was selected to retain genes with potential biological relevance while maintaining optimal model performance, as our primary objective was not only risk prediction but also downstream biological and mechanistic interpretation. LASSO Cox regression was performed with an alpha value of 1 using the glmnet package, and cross-validation was conducted with a fixed random seed (seed = 10403) to ensure reproducibility. The genes with non-zero coefficients under λ_min were subsequently subjected to stepwise multivariable Cox regression to further refine the model. This integrative approach resulted in the construction of a final 28-gene prognostic signature.

### 4.5. Risk Score Calculation and Patient Stratification

A prognostic risk score was calculated for each patient as a weighted sum of expression levels of the 28 genes, with weights corresponding to their Cox regression coefficients. For primary survival analyses and clinical association testing, patients were dichotomized into high-risk and low-risk groups using the median risk score to ensure balanced group sizes and robust statistical power. To further assess whether the risk score reflected a continuous biological gradient rather than an arbitrary binary cutoff, additional stratification into tertiles (low, intermediate, and high risk) was performed for selected analyses evaluating risk-dependent trends. Baseline clinicopathological characteristics of the dichotomized risk groups are summarized in Table S4, and the multivariable Cox regression model incorporating the 28-gene risk score, FIGO stage, and tumor grade is presented in Table S5.

The prognostic risk score was calculated for each patient as a linear combination of gene expression levels weighted by their corresponding regression coefficients derived from the final Cox model:

Risk score = Σ (βi × expressioni)

where βi represents the regression coefficient of gene i. The full list of coefficients used for risk score calculation is provided in Table S6.

### 4.6. Survival Analysis and Model Evaluation

Kaplan–Meier survival analysis and log-rank tests were used to compare overall survival between risk groups. Univariate and multivariable Cox proportional hazards regression analyses were performed to assess whether the risk score was independently associated with survival after adjustment for clinical covariates, including age.

The proportional hazards assumption of the Cox regression models was evaluated using Schoenfeld residual-based global tests, and no significant violations were detected, supporting the validity of the Cox proportional hazards models applied in this study. Time-dependent ROC curves were generated to evaluate the predictive performance of the 28-gene signature at 1-, 3-, and 5-year time points. Model calibration and discrimination were further assessed using nomogram analysis and area under the curve (AUC) metrics.

To evaluate model robustness at the sample level, sample-level leave-one-out cross-validation (LOO-CV) was performed by iteratively excluding one patient at a time, refitting the prognostic model on the remaining samples, and recalculating the 3-year time-dependent AUC. In addition, leave-one-gene-out sensitivity analysis was conducted as a complementary robustness assessment, in which each gene was sequentially removed from the 28-gene signature and the resulting change in 3-year AUC (ΔAUC) was evaluated to assess the contribution of individual genes to overall model performance.

### 4.7. External Biological and Clinicopathological Validation

An independent microarray dataset (GSE17025; downloaded in January 2026) was used for external validation. After probe-to-gene mapping, only genes from the original 28-gene signature that were available on the GSE17025 platform were retained, resulting in a 19-gene subset for validation analyses. Expression values were standardized at the gene level using z-score normalization to enhance comparability across platforms. Signature activity was calculated using the same z-score-based aggregation strategy as applied in the TCGA cohort, without any cross-cohort model refitting. Associations between signature activity and tumor–normal status, histological subtype, and tumor grade were evaluated using non-parametric statistical tests.

### 4.8. Functional Enrichment and Biological Pathway Analysis

To explore the biological relevance of the 28-gene signature, functional enrichment analyses were performed using the Enrichr web-based platform (https://maayanlab.cloud/Enrichr/; accessed on 22 December 2025). Gene Ontology (GO), Kyoto Encyclopedia of Genes and Genomes (KEGG), Reactome, and Hallmark gene sets were queried to identify biological processes and pathways significantly overrepresented among the signature genes. Enrichment significance was evaluated using the statistical framework implemented in Enrichr, and enriched terms were ranked according to adjusted *p* values. These enrichment analyses were used to provide functional context for the 28-gene signature, whereas downstream transcriptomic signature-based analyses were conducted independently using predefined marker gene sets, rather than enrichment-derived gene lists.

Protein–protein interaction (PPI) analysis was performed using the STRING database (version 12.0). To balance network connectivity and interaction reliability in a relatively small gene set, a confidence score cutoff of 0.300 was applied, which is slightly below the default medium-confidence threshold (0.400) but above low-confidence interactions. This setting allowed inclusion of biologically plausible interactions while avoiding excessive noise from weakly supported associations.

### 4.9. Immune-Related Signature Analysis

To characterize the immune context associated with the prognostic signature, immune-related transcriptomic signature scores were compared between low- and high-risk groups defined by the extreme tertiles of the risk score, a strategy adopted to enhance sensitivity for detecting risk-associated immune transcriptional differences. The analyzed signatures included those reflecting CD8^+^ T cell infiltration, cytotoxic activity (CYT), IFNG signaling, macrophage-associated programs, global T cell signatures, and regulatory T cell (Treg)-related signatures. Group-wise differences were evaluated using the Wilcoxon rank-sum test. In parallel, Spearman correlation analyses were performed to assess associations between continuous risk scores and immune signature scores, as well as to examine the internal coherence among immune activation-related metrics.

Immune cell fractions were estimated using CIBERSORTx (https://cibersortx.stanford.edu/; accessed on 31 December 2025) with the LM22 signature matrix [37]. Gene expression profiles derived from bulk RNA-seq data were used as input, with quantile normalization disabled, as recommended by the developers for RNA-seq-based analyses. To enhance computational stability and reduce noise, the mixture matrix was restricted to genes overlapping with the LM22 reference. Statistical significance was evaluated using 100 permutations. In addition to predefined immune transcriptomic signatures, selected LM22 immune cell subsets, including macrophage polarization states (M0, M1, and M2), neutrophils, dendritic cell states (resting and activated), and NK cell states (resting and activated), were further examined to explore whether risk stratification was associated with specific immune cell compositions. These analyses were conducted in an exploratory manner and are reported in the Supplementary Information, without being used as primary determinants of biological interpretation or mechanistic inference. Accordingly, downstream analyses and biological interpretations primarily focused on immune signatures with established robustness and reproducibility in bulk transcriptomic datasets, particularly T cell-related and cytotoxic immune axes.

### 4.10. Transcriptomic Signature Analysis of Tumor-Associated Biological Programs

To characterize tumor-intrinsic and microenvironment-associated biological programs, curated transcriptomic signatures were quantified using a marker-based scoring approach. All signature scores were calculated at the individual-sample level based on normalized gene expression values.

Epithelial–mesenchymal transition (EMT) activity was quantified using a marker-based scoring approach by integrating the expression of mesenchymal/extracellular matrix (ECM)-associated genes (*VIM*, *FN1*, *COL1A1*, *COL3A1*, and *SPARC*) and epithelial markers (*CDH1* and *EPCAM*). Specifically, the EMT score for each sample was defined as the mean expression of mesenchymal/ECM-associated genes minus the mean expression of epithelial markers. This formulation provides a quantitative index of EMT activity by contrasting mesenchymal/ECM-associated transcriptional programs with epithelial identity.

Additional tumor behavior-related programs were evaluated using curated marker gene sets, with signature scores calculated as the average expression of the corresponding genes within each sample. These programs included angiogenesis (*VEGFA*, *VEGFB*, *VEGFC*, *KDR*, *FLT1*, and *ANGPT2*), cell migration/invasion (*MMP2*, *MMP9*, *ITGA5*, *ITGB1*, *CXCR4*, and *S100A4*), ECM remodeling (*COL1A1*, *COL3A1*, *FN1*, and *SPARC*), hypoxia (*HIF1A*, *CA9*, *VEGFA*, and *LDHA*), cell cycle regulation (*MKI67*, *CCNB1*, and *TOP2A*), DNA repair (*BRCA1*, *RAD51*, and *CHEK1*), drug resistance (*ABCB1*, *ABCC1*, and *ABCG2*), and oxidative stress (*NFE2L2*, *SOD1*, *GPX1*, and *CAT*).

All signature scores were compared between risk groups using the Wilcoxon rank-sum test. Where applicable, multiple testing correction was performed using the Benjamini–Hochberg method. These marker genes were selected based on established literature and commonly used transcriptomic definitions of each biological program.

### 4.11. Overall Study Design and Analytical Workflow

To systematically identify a prognostic gene signature for UCEC, we established an integrative analytical framework based on transcriptome-wide expression profiles from the TCGA-UCEC cohort (Figure 1). Baseline clinicopathological characteristics of the analyzed patients are summarized in Table S4. Normalized RNA-seq data were analyzed using a two-step screening strategy. First, stage-based differential expression analysis was performed to identify genes associated with disease progression by comparing early-stage and late-stage tumors. In parallel, survival-associated genes were identified using univariate Cox proportional hazards regression based on overall survival. Genes identified from both analyses were intersected to generate a set of candidate genes with combined biological relevance and prognostic potential. These candidates were further evaluated using univariate Cox regression, followed by LASSO Cox regression with 10-fold cross-validation to reduce overfitting. The optimal penalty parameter was selected based on cross-validation criteria, and the stability of gene coefficients across penalization paths was examined (Figure S2). The resulting gene set was used to construct a multigene prognostic model, which was subsequently subjected to downstream validation, functional characterization, and robustness analyses as described below.

### 4.12. Statistical Analysis

All statistical analyses were conducted using R software (version 4.5.2). Two-sided *p*-values < 0.05 were considered statistically significant unless otherwise stated. Multiple testing corrections were applied where appropriate. Data visualization was performed using ggplot2 and related packages.

### 4.13. Use of AI-Assisted Tools

AI-assisted language models were used for language editing, clarity enhancement, and image optimization during manuscript preparation. Specifically, ChatGPT (OpenAI, GPT-5.5 version) was used to improve the grammar, coherence, and readability of the text. Additionally, AI-assisted image enhancement tools were employed to improve the visual clarity and resolution of the data plots to address formatting requirements. These tools did not contribute to the study design, raw data analysis, statistical modeling, or the interpretation of results. The authors confirm that the AI-assisted image optimization did not alter the underlying research data or the integrity of the original images.

## Figures and Tables

**Table 1:** Univariate Cox analysis of individual genes in the 28-gene signature. Univariate Cox regression analysis of individual genes included in the 28-gene prognostic signature. For each gene, regression coefficients (β), hazard ratios (HRs), log2-transformed HRs, −log10(*p* values), and corresponding statistical significance are reported.

Gene	Beta	HR	*p*	log2HR	neglog10p
*FP565260.3*	0.959	2.61	1.11 × 10^−4^	1.383	3.955
*GFRA4*	0.022	1.02	2.26 × 10^−5^	0.031	4.645
*LINC00683*	−1.783	0.17	1.13 × 10^−3^	−2.572	2.948
*DDC*	0.057	1.06	3.68 × 10^−6^	0.083	5.434
*LRRIQ4*	0.281	1.32	4.79 × 10^−4^	0.406	3.320
*KLRG2*	0.112	1.12	2.90 × 10^−3^	0.162	2.538
*UNQ6494*	−5.445	0.00	2.09 × 10^−3^	−7.855	2.680
*STAC*	0.871	2.39	2.11 × 10^−12^	1.257	11.675
*LINC00475*	−2.509	0.08	0.034	−3.620	1.467
*AC073210.3*	0.143	1.15	8.75 × 10^−3^	0.207	2.058
*CHODL*	0.035	1.04	8.28 × 10^−3^	0.050	2.082
*AC012354.9*	0.978	2.66	6.18 × 10^−5^	1.411	4.209
*AL121906.2*	0.199	1.22	1.78 × 10^−4^	0.287	3.749
*AC011995.2*	4.314	74.73	1.60 × 10^−4^	6.224	3.797
*ARX*	0.015	1.02	0.197	0.022	0.706
*RNF2P1*	1.599	4.95	3.99 × 10^−6^	2.307	5.399
*AL359313.1*	1.505	4.50	1.07 × 10^−4^	2.171	3.971
*AC010729.2*	2.524	12.48	3.91 × 10^−6^	3.641	5.407
*CTAGE11P*	6.683	798.32	1.57 × 10^−4^	9.641	3.805
*OSTN*	0.580	1.79	0.056	0.837	1.251
*MAFA*	0.050	1.05	0.094	0.071	1.029
*CDK5R2*	0.083	1.09	3.69 × 10^−6^	0.120	5.433
*CAMKV*	0.183	1.20	0.024	0.264	1.614
*TTPA*	0.344	1.41	0.015	0.496	1.837
*NOL4*	0.206	1.23	4.26 × 10^−4^	0.297	3.370
*EN1*	0.107	1.11	0.017	0.154	1.774
*NSG2*	0.076	1.08	2.64 × 10^−7^	0.109	6.578
*CDH18*	0.136	1.15	2.69 × 10^−5^	0.197	4.570

**Table 2:** Transcript-level associations of PPI-identified hub genes with the 28-gene risk score and overall survival in the TCGA-UCEC cohort. The table summarizes Spearman correlation coefficients between gene expression and the 28-gene risk score, Wilcoxon rank-sum test *p* values comparing high- and low-risk groups, and univariate Cox proportional hazards regression results, including hazard ratios (HRs), 95% confidence intervals (CIs), and corresponding *p* values.

Gene	Spearman Rho	Spearman *p*	Wilcoxon *p*	HR	CI 95	Cox *p*
*EZH2*	0.286	9.11 × 10^−12^	1.69 × 10^−10^	1.061	1.023–1.1	1.46 × 10^−3^
*CDK5*	−0.067	0.118	0.15	1.015	0.969–1.062	0.535
*CDK5R1*	0.071	0.096	0.363	1.142	1.029–1.267	0.012
*CDK5R2*	0.522	9.13 × 10^−40^	7.88 × 10^−24^	1.087	1.049–1.126	3.69 × 10^−6^
*MAP3K1*	0.036	0.406	0.95	0.983	0.941–1.027	0.447

## Data Availability

The datasets analyzed in this study are publicly available. RNA sequencing data and corresponding clinical information for uterine corpus endometrial carcinoma (UCEC) were obtained from The Cancer Genome Atlas (TCGA) data portal (https://portal.gdc.cancer.gov/; accessed on 6 November 2025). The external validation dataset was retrieved from the Gene Expression Omnibus (GEO) database under accession number GSE17025. All data used in this study are publicly accessible and were downloaded in accordance with the relevant data access policies.

## Supplementary Materials

The following supporting information can be downloaded at: https://www.mdpi.com/article/10.3390/ijms27104170/s1.

## Abbreviations

UCEC	Uterine corpus endometrial carcinoma
TCGA	The Cancer Genome Atlas
GEO	Gene Expression Omnibus
DEGs	Differentially expressed genes
LOO	Leave-one-out
LM22	Leukocyte gene signature matrix (22 immune cell types)
EMT	Epithelial–mesenchymal transition
