# Supplementary material for: Transcriptomic Evidence of Immune–Tumor Uncoupling Defines a High-Risk State in Uterine Corpus Endometrial Carcinoma
Source: Int J Mol Sci. 2026 May 7;27(10):4170. doi: 10.3390/ijms27104170 (PMC13208045; doi:10.3390/ijms27104170)
Supplement: Supplementary file 1 [file ijms-27-04170-s001.zip › Supplementary information_Proofreading.pdf]

**Supplementary results**

**Table S1. Age-adjusted Cox regression analysis for overall survival.** Multivariable Cox proportional hazards analysis evaluating the prognostic value of the 28-gene risk score after adjustment for age. Hazard ratios (HRs), 95% confidence intervals (CIs), and *p* values are presented.

| Term | HR | CI low | CI high | *p* |
|---|---|---|---|---|
| **Risk score lasso** | 7.783 | 5.024 | 12.057 | 4.00E-20 |
| **Age** | 1.034 | 1.012 | 1.057 | 0.002 |

**Table S2. Leave-one-gene-out sensitivity analysis of individual genes within the 28-gene signature.** Results of leave-one-gene-out analysis evaluating the impact of removing each gene on the 3-year AUC of the prognostic model. The change in AUC (ΔAUC) reflects the contribution of each gene to overall model performance. No single gene removal resulted in a dramatic loss of predictive accuracy, supporting the stability and distributed nature of the signature.

| **Gene_removed** | **Ensembl_ID** | **AUC_3yr** | **delta_AUC** |
|---|---|---|---|
| *LINC01431* | ENSG00000277117 | 0.805 | -6.45E-03 |
| *GFRA4* | ENSG00000125861 | 0.807 | -4.60E-03 |
| *AL583794.1* | ENSG00000266256 | 0.808 | -4.44E-03 |
| *DDC* | ENSG00000132437 | 0.808 | -4.43E-03 |
| *SPARC* | ENSG00000188306 | 0.808 | -3.47E-03 |
| *KLRG2* | ENSG00000188883 | 0.809 | -3.35E-03 |
| *AL365204.2* | ENSG00000237372 | 0.809 | -2.92E-03 |
| *STAC* | ENSG00000144681 | 0.809 | -2.79E-03 |
| *LINC02482* | ENSG00000225511 | 0.810 | -2.36E-03 |
| *AL353683.1* | ENSG00000286342 | 0.810 | -2.31E-03 |
| *CHODL* | ENSG00000154645 | 0.810 | -2.14E-03 |
| *AL359051.1* | ENSG00000278957 | 0.810 | -1.96E-03 |
| *LINC01138* | ENSG00000275223 | 0.810 | -1.64E-03 |
| *EPAS1* | ENSG00000237720 | 0.811 | -1.30E-03 |
| *ARX* | ENSG00000004848 | 0.811 | -1.29E-03 |
| *NFE2L2* | ENSG00000231381 | 0.811 | -8.51E-04 |
| *SIX2* | ENSG00000236366 | 0.811 | -6.18E-04 |
| *AC010729.1* | ENSG00000242540 | 0.812 | -4.33E-04 |
| *AP000305.1* | ENSG00000214249 | 0.812 | -4.24E-04 |
| *OSTN* | ENSG00000188729 | 0.812 | 5.85E-05 |
| *MAFA* | ENSG00000182759 | 0.812 | 1.41E-04 |
| *CDK5R2* | ENSG00000171450 | 0.812 | 2.28E-04 |
| *CAMKV* | ENSG00000164076 | 0.813 | 7.26E-04 |
| *CCND1* | ENSG00000137561 | 0.813 | 1.23E-03 |

| | | | |
|---|---|---|---|
| *NOL4* | ENSG00000101746 | 0.813 | 1.47E-03 |
| *EN1* | ENSG00000163064 | 0.814 | 2.35E-03 |
| *POU5F1* | ENSG00000170091 | 0.815 | 3.19E-03 |
| *CDH18* | ENSG00000145526 | 0.820 | 7.83E-03 |

**Table S3. Functional annotation of genes included in the 28-gene prognostic signature.** Table S3 summarizes the constituent genes of the 28-gene prognostic signature along with concise descriptions of their reported biological functions. Gene annotations were curated from publicly available databases and literature, with a focus on established roles in tumor biology, including immune regulation, cell cycle control, transcriptional regulation, signal transduction, metabolic adaptation, and stress response. This table provides functional context for interpreting the biological themes captured by the prognostic signature and supports downstream pathway, immune, and protein–protein interaction analyses.

| Gene Symbol | Concise functional annotation |
|---|---|
| *LINC01431* | Long non-coding RNA involved in transcriptional regulation. |
| *GFRA4* | Cell-surface receptor of the GDNF family mediating RET signaling and regulating cell survival and differentiation. |
| *AL583794.1* | Non-coding RNA potentially involved in transcriptional or chromatin-level regulation. |
| *DDC* | Enzyme catalyzing the final steps of dopamine and serotonin biosynthesis, involved in cellular metabolic and signaling processes. |
| *SPARC* | Secreted matricellular protein regulating extracellular matrix remodeling and cell–matrix interactions. |
| *KLRG2* | C-type lectin-like receptor involved in immune cell signaling and regulation. |
| *AL365204.2* | Non-coding RNA with potential regulatory roles in gene expression. |
| *STAC* | Adaptor protein involved in intracellular calcium signaling and signal transduction. |
| *LINC02482* | Long non-coding RNA potentially involved in transcriptional regulation. |
| *AL353683.1* | Non-coding RNA with putative regulatory functions. |
| *CHODL* | Cell surface protein involved in cell adhesion and extracellular matrix interactions. |

| | |
|---|---|
| *AL359051.1* | Non-coding RNA potentially involved in transcriptional regulation. |
| *LINC01138* | Long non-coding RNA implicated in regulation of cell proliferation and gene expression. |
| *EPAS1* | Transcription factor regulating hypoxia-responsive genes and angiogenic signaling. |
| *ARX* | Homeobox transcription factor involved in developmental regulation and cell fate determination. |
| *NFE2L2* | Transcription factor controlling oxidative stress response and cellular redox homeostasis. |
| *SIX2* | Homeobox transcription factor involved in developmental programs and cellular differentiation. |
| *AC010729.1* | Non-coding RNA with potential roles in transcriptional regulation. |
| *AP000305.1* | Non-coding RNA potentially involved in gene regulatory processes. |
| *OSTN* | Secreted protein involved in extracellular signaling and tissue homeostasis. |
| *MAFA* | Transcription factor regulating cellular differentiation and gene expression programs. |
| *CDK5R2* | Regulatory subunit of CDK5 involved in cell cycle–related and neuronal signaling pathways. |
| *CAMKV* | Kinase-like protein associated with calcium/calmodulin-mediated signaling. |
| *CCND1* | Cell cycle regulator controlling G1/S phase transition and cellular proliferation. |
| *NOL4* | Nucleolar protein potentially involved in transcriptional regulation and cell cycle processes. |
| *EN1* | Homeobox transcription factor involved in developmental patterning and transcriptional regulation. |
| *POU5F1* | Pluripotency-associated transcription factor regulating stemness and cell fate. |
| *CDH18* | Cadherin family cell adhesion molecule involved in cell–cell interactions. |

**Table S4. Baseline clinical characteristics of patients stratified by the 28-gene risk score.** Patients were divided into low- and high-risk groups according to the median 28-gene risk score. Values are presented as median (IQR) or number (percentage), as appropriate. Missing or unavailable values are indicated where applicable. OS, overall survival; FIGO, International Federation of Gynecology and Obstetrics.

| Variable | Low | High |
|---|---|---|
| Age, years, median (IQR) | 61.0 (55.0–69.7) | 67.3 (61.0-73.5) |
| OS events, n (%) | 17 (6.3%) | 73 (26.9%) |
| OS time, median days | 994 | 820 |
| Missing data, n | 25 | 59 |
| FIGO stage | | |
| Stage I | 3 (1.1%) | 1 (0.4%) |
| Stage IA | 97 (35.9%) | 49 (18.1%) |
| Stage IB | 77 (28.5%) | 55 (20.3%) |
| Stage IC | 11 (4.1%) | 9 (3.3%) |
| Stage II | 12 (4.4%) | 15 (5.5%) |
| Stage IIA | 1 (0.4%) | 5 (1.8%) |
| Stage IIB | 5 (1.9%) | 5 (1.8%) |
| Stage III | 0 (0%) | 2 (0.7%) |
| Stage IIIA | 14 (5.2%) | 16 (5.9%) |
| Stage IIIB | 4 (1.5%) | 2 (0.7%) |
| Stage IIIC | 9 (3.3%) | 20 (7.4%) |
| Stage IIIC1 | 7 (2.6%) | 10 (3.7%) |
| Stage IIIC2 | 5 (1.9%) | 11 (4.1%) |
| Stage IV | 1 (0.4%) | 3 (1.1%) |
| Stage IVA | 0 (0%) | 2 (0.7%) |
| Stage IVB | 3 (1.1%) | 11 (4.1%) |
| NA | 20 (7.4%) | 55 (20.3%) |
| Stage IB1 | 1 (0.4%) | 0 (0%) |
| Tumor grade | | |
| G1 | 79 (29.3%) | 13 (4.8%) |
| G2 | 80 (29.6%) | 26 (9.6%) |

| | | |
|---|---|---|
| G3 | 87 (32.2%) | 168 (62%) |
| High Grade | 2 (0.7%) | 8 (3%) |
| NA | 22 (8.1%) | 56 (20.7%) |
| Histology | | |
| Endometrioid | 201 (74.4%) | 98 (36.2%) |
| Mixed | 3 (1.1%) | 10 (3.7%) |
| Serous | 5 (1.9%) | 48 (17.7%) |
| Missing/Unknown | 61 (22.6%) | 115 (42.4%) |

**Table S5. Multivariable Cox regression analysis for overall survival including the 28-gene risk score, FIGO stage, and tumor grade.** The model included the continuous 28-gene risk score, FIGO stage group, and tumor grade. FIGO stage was modeled as III–IV versus I–II, and tumor grade was modeled as G3 versus G1–G2. Hazard ratios (HRs), 95% confidence intervals (CIs), *p* values, sample size (N), and number of overall survival events are shown.

| Variable | HR | CI | *p* value | N | Events |
|---|---|---|---|---|---|
| **28-gene risk score** | 6.434 | 3.865-10.709 | 8.04E-13 | 549 | 92 |
| **FIGO stage III-IV vs I-II** | 2.654 | 1.735-4.058 | 6.68E-06 | 549 | 92 |
| **Tumor grade G3 vs G1-G2** | 1.72 | 0.962-3.073 | 0.0673 | 549 | 92 |

**Table S6. Final coefficients used for calculation of the 28-gene risk score.** This table lists the Ensembl gene identifiers, corresponding gene symbols, and regression coefficients (β) derived from the LASSO Cox model (λmin) used to construct the prognostic risk score. The risk score for each patient was calculated as a linear combination of gene expression levels weighted by these coefficients.

| **Gene_Symbol** | **Ensembl_ID** | **Coefficient** |
|---|---|---:|
| *AC012354.9* | ENSG00000278957 | 0.097835183 |
| *EN1* | ENSG00000163064 | 0.048787353 |
| *LRRIQ4* | ENSG00000188306 | 0.044688343 |
| *NSG2* | ENSG00000170091 | 0.039982693 |
| *AC010729.2* | ENSG00000242540 | 0.039547082 |
| *TTPA* | ENSG00000137561 | 0.03180513 |
| *FP565260.3* | ENSG00000277117 | 0.028398439 |
| *UNQ6494* | ENSG00000237372 | -0.023192721 |
| *CHODL* | ENSG00000154645 | 0.023053197 |
| *CDH18* | ENSG00000145526 | 0.02086208 |
| *CTAGE11P* | ENSG00000214249 | 0.01662728 |
| *DDC* | ENSG00000132437 | 0.016423642 |
| *GFRA4* | ENSG00000125861 | 0.014388781 |
| *ARX* | ENSG00000004848 | 0.014275831 |
| *AC073210.3* | ENSG00000286342 | 0.013879467 |
| *KLRG2* | ENSG00000188883 | 0.011632943 |
| *CDK5R2* | ENSG00000171450 | 0.011104245 |
| *NOL4* | ENSG00000101746 | 0.010972543 |
| *CAMKV* | ENSG00000164076 | 0.010533252 |
| *LINC00475* | ENSG00000225511 | -0.010311552 |
| *AC011995.2* | ENSG00000237720 | 0.010309787 |
| *RNF2P1* | ENSG00000231381 | 0.00926786 |
| *LINC00683* | ENSG00000266256 | -0.007870587 |
| *STAC* | ENSG00000144681 | 0.007652523 |
| *AL121906.2* | ENSG00000275223 | 0.006952645 |
| *AL359313.1* | ENSG00000236366 | 0.005095312 |

| | | |
|---|---|---|
| *OSTN* | ENSG00000188729 | 0.003041361 |
| *MAFA* | ENSG00000182759 | 0.001008353 |

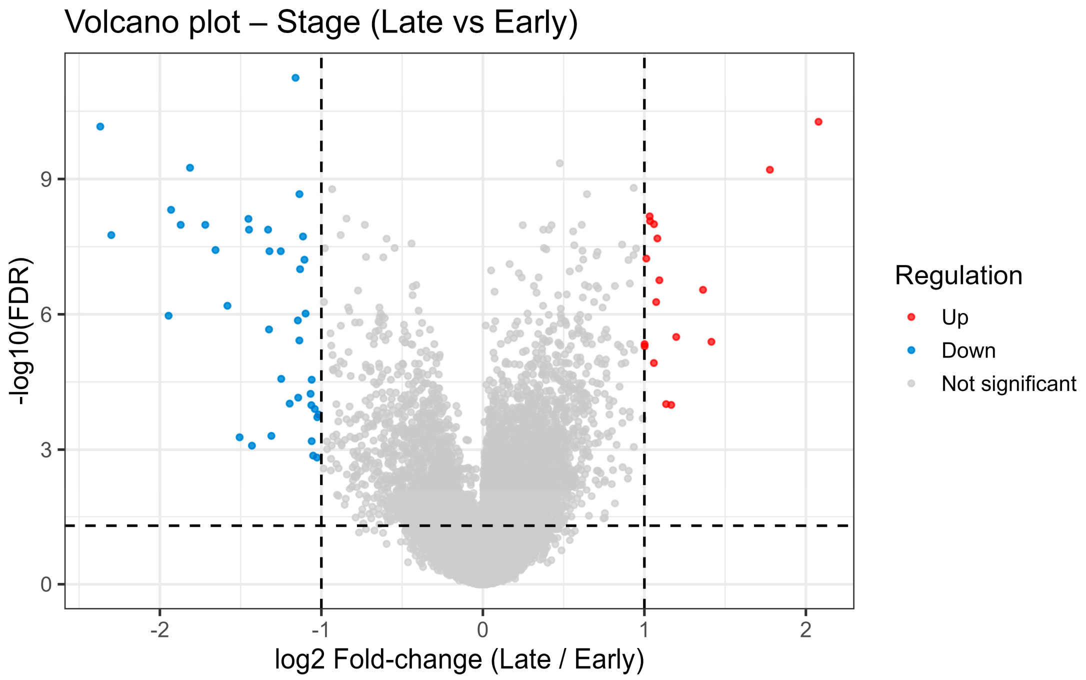


**Figure S1. Identification of stage-associated differentially expressed genes in TCGA-UCEC.** Volcano plot illustrates differential gene expression between late-stage and early-stage UCEC tumors. The x-axis represents log2 fold change (late-stage versus early-stage), and the y-axis represents −log10 false discovery rate (FDR). Vertical dashed lines indicate the predefined fold-change threshold ($|\log2FC| \geq 1$), and the horizontal dashed line indicates the FDR significance cutoff ($FDR < 0.05$). Genes significantly upregulated in late-stage tumors are shown in red, genes downregulated in late-stage tumors are shown in blue, and non-significant genes are shown in gray. These stage-associated differentially expressed genes were subsequently integrated with survival-associated genes as part of the multi-step candidate gene selection strategy.

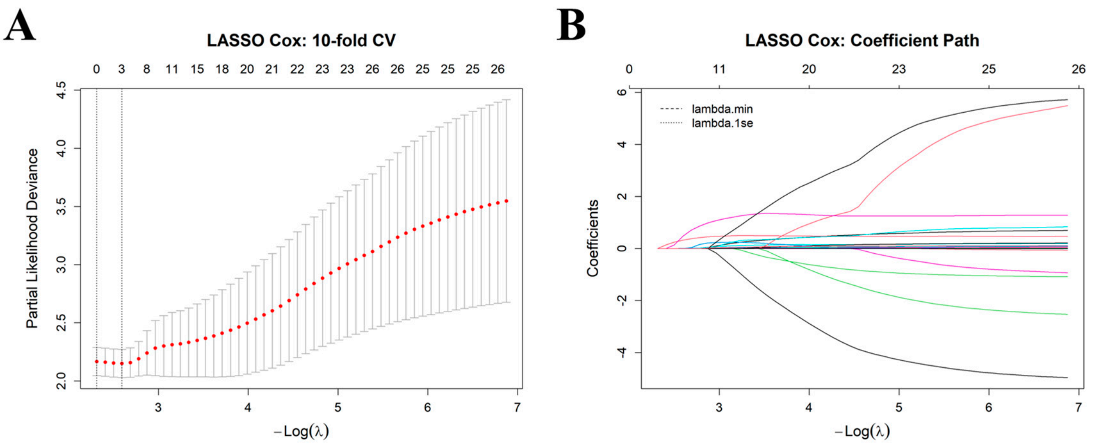


**Figure S2. Construction of the prognostic gene signature using LASSO Cox regression. (A)** Ten-fold cross-validation curve for the LASSO Cox proportional hazards model, showing the partial likelihood deviance as a function of the regularization parameter (log λ). The optimal value of λ was selected based on the minimum cross-validated error criterion. The final signature was derived from non-zero coefficients at λ_min. **(B)** Coefficient paths of candidate genes in the LASSO Cox regression model as a function of log λ. Each curve represents the trajectory of a gene coefficient as the penalty strength increases, with a subset of genes retaining non-zero coefficients at the optimal λ, resulting in the final 28-gene prognostic signature.

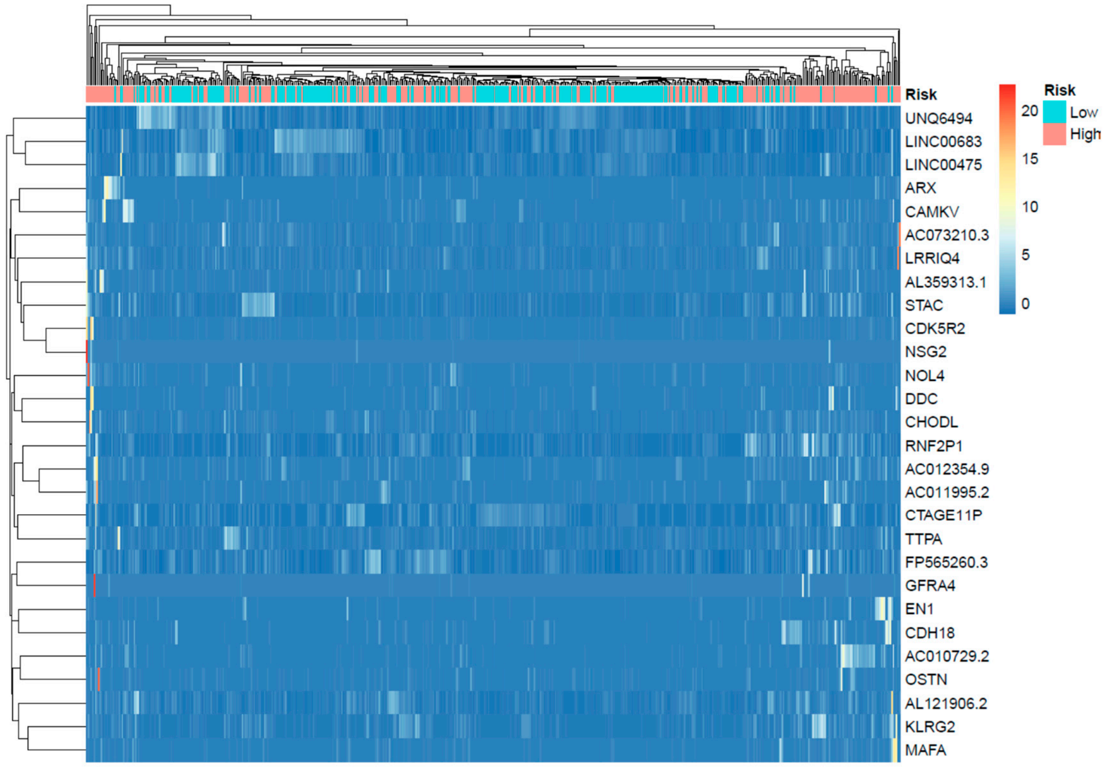


**Figure S3. Expression landscape of the 28-gene prognostic signature across risk groups.** Heatmap showing the expression profiles of the 28 genes constituting the prognostic signature across the TCGA-UCEC cohort. Samples are ordered by increasing risk score and annotated according to risk group (low vs high). Gene expression values were normalized and scaled by genes to facilitate comparison across samples. Unsupervised hierarchical clustering was applied to both genes and samples, illustrating distinct expression patterns associated with prognostic risk stratification.

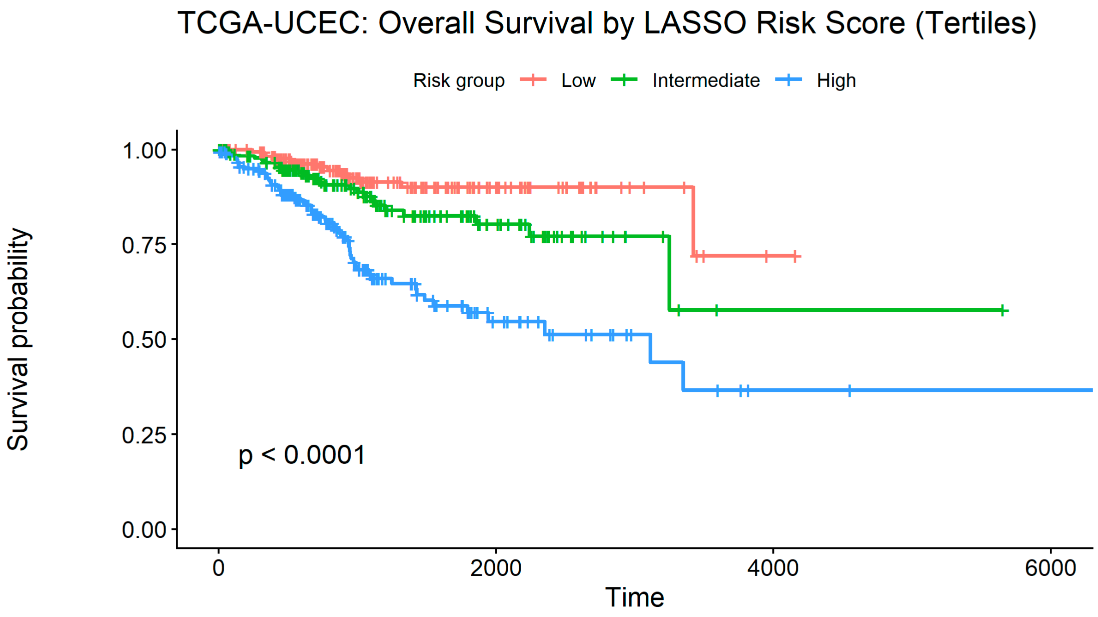


**Figure S4. Overall survival stratification by tertiles of the LASSO-derived risk score.** Kaplan–Meier overall survival (OS) curves for patients in the TCGA-UCEC cohort stratified into low-, intermediate-, and high-risk groups according to tertiles of the 28-gene LASSO-derived risk score. A significant stepwise decrease in overall survival was observed with increasing risk score (log-rank test, $p < 0.0001$), indicating a dose-dependent association between the risk score and patient prognosis.

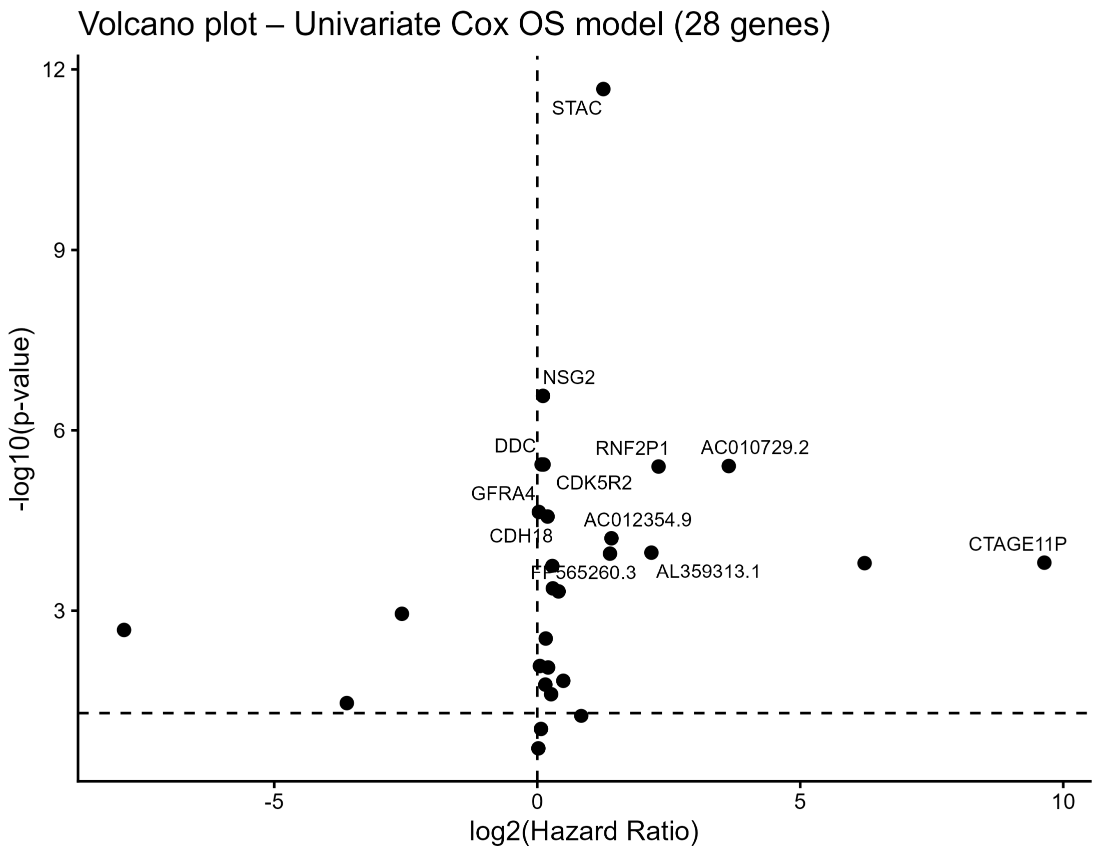


**Figure S5. Univariate prognostic effects of individual genes within the 28-gene signature.** Volcano plot summarizes univariate Cox regression results for individual genes included in the 28-gene prognostic signature. The x-axis represents log2 hazard ratios, and the y-axis shows −log10(*p* values). The vertical dashed line indicates a hazard ratio of 1 (log2 HR = 0), and the horizontal dashed line denotes the nominal significance threshold (*p* = 0.05). While several genes exhibit statistically significant associations with overall survival, effect sizes vary across individual components, supporting the rationale for integrating multiple genes into a composite prognostic model rather than relying on single-gene predictors.

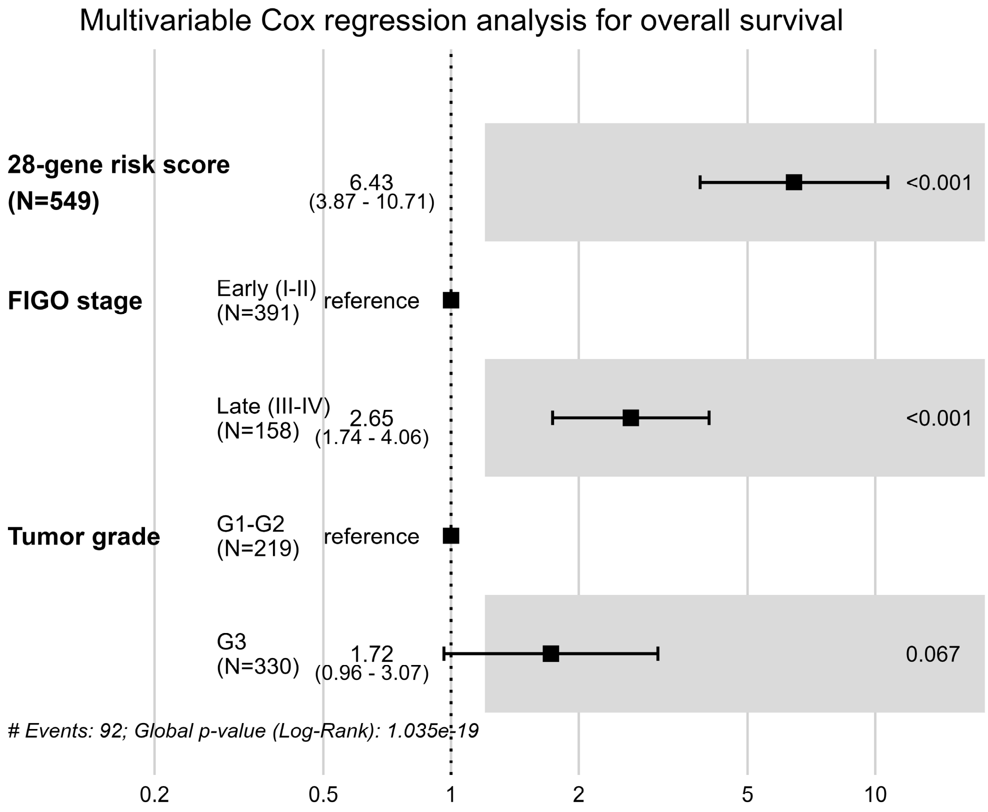


**Figure S6. Multivariable Cox regression analysis incorporating clinical covariates.**

Multivariable Cox proportional hazards regression analysis for overall survival including the 28-gene LASSO-derived risk score and established clinicopathological variables (FIGO stage and tumor grade). Hazard ratios (HRs) with 95% confidence intervals are shown, and *p* values were derived from Wald tests. The analysis demonstrates that the 28-gene risk score remains an independent prognostic factor after adjustment for conventional clinical parameters.
